# A body composition-based clustering study and its association with metabolic phenotypes among the general population in China

**DOI:** 10.3389/fnut.2025.1636849

**Published:** 2025-10-31

**Authors:** Dan Xiang, Li Yuan, Weimin Chen, Yan Wu, Yangtian Wang

**Affiliations:** ^1^Department of Endocrinology, Taikang Xianlin Drum Tower Hospital, Affiliated Hospital of Medical School, Nanjing University, Nanjing, China; ^2^Department of Health Management Center, Taikang Xianlin Drum Tower Hospital, Affiliated Hospital of Medical School, Nanjing University, Nanjing, China

**Keywords:** metabolic phenotypes, metabolic diseases, serum lipid levels, obese population, risk, body composition

## Abstract

**Objective:**

Metabolic phenotypes are linked to various metabolic diseases, but current classification methods have limitations. This study aims to directly cluster obese populations based on body composition and machine learning, enhancing understanding of lipid metabolism and disease associations.

**Methods:**

A retrospective analysis included participants who underwent InBody examinations at Taikang Xianlin Drum Tower Hospital in 2023. Subjects were categorized into four phenotypes: MHNW, MHO, MUNW, and MUO, based on BMI and metabolic syndrome criteria. Correlations between InBody indexes and clinical data were analyzed. Machine learning cluster analysis identified subgroupings, and associations with metabolic diseases were examined.

**Results:**

InBody indexes correlated strongly with medical history and lab results. Clustering classified males into two groups and females into three, with significant differences in age, weight, height, BMI, and InBody scores (all *p* < 0.001). The prevalence of hypertension and hyperlipidemia varied notably among male subgroups, while hypertension and diabetes showed significant differences among female subgroups.

**Conclusion:**

The InBody-based clustering analysis showed males could be categorized into 2 subgroups while females could be classified into 3 subgroups, indicating that the population with a specific InBody clustering profile could be at higher risk of metabolic diseases.

## Introduction

In recent years, with the rapid development of economics, obesity and overweight are becoming more prevalent and are currently two of the most common health and economic challenges (1). Moreover, several complications have been identified to have a strong association with overweight and obesity, including metabolic syndromes, cardiovascular disease, hypertension, and lung disease (2–5). However, there are many unhealthy people with normal weight and healthy people with increased body weight, therefore, many studies have pointed out the limitation of using solely overweight or body mass index (BMI) as risk factors, and metabolic phenotype has been proposed for better evaluation of people’s health status (6).

Body composition has been reported to be associated with various diseases, including obesity (7), diabetes (8), hypertension (9), and hyperurecemia (10). Therefore, accurate estimation of body composition is of great importance and in urgent need. Currently, dual-energy X-ray absorptiometry (DEXA), computed tomography (CT), and magnetic resonance imaging (MRI) are considered the gold standard of body component estimation (11, 12). However, the application of previously mentioned radiological examinations in the clinical scenario is limited by their inconvenience, and the presence of the bioimpedance analysis (BIA) method and the portable InBody equipment provides an effective replacement option for the traditional radiological tools. More importantly, the reliability and repeatability of BIA methods are widely validated in different cohorts and various BIA machines, which makes them more acceptable for clinical usage (13, 14). The outbreak of body composition data also facilitates the health assessment, Xia et al. showed that increased adiposity and decreased skeletal muscle mass are associated with desirable metabolic traits in Chinese adults with normal weight (15), Deng et al. also used anthropometric indices as tools for evaluating health (16). Those studies indicating that InBody indicators could be potential biomarkers compared to traditional indices.

Cluster analysis, as a machine learning-based method, has gained increasing popularity in the past few decades, and it could provide a new perspective for the understanding of populations with different traits. Ye proposed a K-means cluster method for the improved discrimination of newly-onset metabolic-associated fatty liver disease (17); Takeshita et al. clustered obesity patients into seven subgroups and indicated that some metabolically healthy obesity groups may require interventions (18). However, to the best of our knowledge, there is no study focused on cluster analysis based on body composition and related to metabolic phenotypes, which may provide new insight for the management of patients with different metabolic phenotypes.

Therefore, this study aims to analyze the performance of cluster analysis based on the InBody indexes in normal weight and obese participants, exploring the underlying relationship between cluster results and participants’ characteristics for the improved understanding of metabolic phenotypes in the Chinese population.

## Materials and methods

### Study population

In this retrospective study, subjects were drawn from the Hospital who visited the Health Management Center between January and December 2023. The inclusion criteria were: (1) patients who completed the InBody test; (2) age between 18 and 75 years. The exclusion criteria were patients without complete clinical data. This study was approved by the Ethics Committee of our hospital (Approval Number: LS202319), and informed consents were waived due to the retrospective nature of this study.

### Data collection and definition

The demographic, hematological, medical history, and InBody test indices were collected from the medical history system. The demographic information, including age, sex, weight, height, systolic blood pressure, diastolic blood pressure, pulse rate, and body mass index, was calculated as weight/height^2^ (kg/m^2^). The medical history includes hypertension, diabetes, hyperlipidemia, coronary artery disease (CAD), coronary artery surgery, hyperuricemia, gout, urolithiasis, nephritis or nephrotic syndrome, diabetic nephropathy, hypothyroidism, fatty liver, and bone mineral density status. While the InBody test indexes include fat fraction (FF), visceral fat area (VFA), basal metabolic rate (BMR), Skeletal Muscle Mass Index (SMI), Fat-Free Mass Index (FFMI), Fat Mass Index (FMI), and the InBody score. The complete hematological data are shown in Table 1.

**Table 1:** Baseline characteristics of participants.

Metabolic phenotypes	MHNW (*N* = 678)	MHO (*N* = 746)	MUNW (*N* = 270)	MUO (*N* = 1,022)	*p*
Male	361 (53.2%)	619 (83.0%)	193 (71.5%)	881 (86.2%)	**<0.001**
Age, years	51.0 [40.0; 58.0]	53.0 [44.0; 60.0]	57.0 [50.0; 64.0]	54.0 [47.0; 60.0]	**<0.001**
Height, cm	167.0 [161.0; 173.0]	170.5 [165.0; 175.0]	169.0 [162.0; 174.0]	171.0 [166.0; 175.0]	**<0.001**
Weight, kg	61.2 [55.8; 66.5]	76.3 [70.7; 82.1]	63.5 [58.0; 68.6]	78.9 [72.6; 85.9]	**<0.001**
BMI, kg/m^2^	22.1 [20.9; 23.0]	26.1 [25.0; 27.6]	22.6 [21.7; 23.3]	27.0 [25.5; 28.9]	**<0.001**
FF, %	26.2 [21.7; 31.0]	29.2 [25.9; 33.5]	26.2 [22.7; 30.1]	30.8 [27.5; 34.5]	**<0.001**
VFA, cm^2^	69.6 [58.9; 83.8]	98.8 [84.7; 122.8]	74.7 [65.0; 88.7]	110.3 [92.6; 134.5]	**<0.001**
BMR, kcal	1346.0 [1208.0; 1482.0]	1548.5 [1436.0; 1652.0]	1394.0 [1250.0; 1499.0]	1563.0 [1449.0; 1674.0]	**<0.001**
SMI, kg/m^2^	6.7 [6.0; 7.4]	7.9 [7.5; 8.4]	7.0 [6.3; 7.4]	8.0 [7.5; 8.5]	**<0.001**
FFMI kg/m^2^	16.0 ± 1.5	18.6 ± 1.6	16.4 ± 1.3	18.9 ± 1.7	**<0.001**
FMI kg/m^2^	5.7 [4.7; 6.8]	7.7 [6.7; 9.1]	5.9 [5.0; 6.9]	8.3 [7.2; 9.7]	**<0.001**
InBody score	70.0 [68.0; 73.0]	68.0 [65.0; 73.0]	69.0 [66.0; 72.0]	67.0 [63.0; 71.0]	**<0.001**
Hypertension	52 (7.7%)	169 (22.7%)	81 (30.0%)	381 (37.3%)	**<0.001**
Diabetes	20 (2.9%)	32 (4.3%)	48 (17.8%)	137 (13.4%)	**<0.001**
Hyperlipidemia	15 (2.2%)	40 (5.4%)	20 (7.4%)	102 (10.0%)	**<0.001**
CAD	3 (0.4%)	12 (1.6%)	4 (1.5%)	17 (1.7%)	0.089
Coronary artery surgery	1 (0.1%)	3 (0.4%)	1 (0.4%)	2 (0.2%)	0.606
Hyperuricemia	6 (0.9%)	25 (3.4%)	6 (2.2%)	36 (3.5%)	**0.006**
Gout	3 (0.4%)	11 (1.5%)	2 (0.7%)	15 (1.5%)	0.153
Urolithiasis	3 (0.4%)	8 (1.1%)	1 (0.4%)	4 (0.4%)	0.321
Nephritis or nephrotic syndrome	2 (0.3%)	0 (0.0%)	0 (0.0%)	0 (0.0%)	0.176
Diabetic nephropathy	0 (0.0%)	0 (0.0%)	0 (0.0%)	0 (0.0%)	-
Hypothyroidism	2 (0.3%)	4 (0.5%)	3 (1.1%)	4 (0.4%)	0.384
SBP	116.0 [106.0; 125.0]	121.0 [113.0; 129.0]	133.0 [121.0; 142.0]	134.0 [125.0; 145.0]	**<0.001**
DBP	68.0 [62.0; 76.0]	74.0 [67.0; 81.0]	78.0 [71.0; 85.0]	81.0 [74.0; 88.0]	**<0.001**
PR	75.0 [69.0; 81.0]	72.0 [66.0; 79.0]	75.0 [69.0; 83.0]	76.0 [70.0; 84.0]	**<0.001**
Fatty liver	139 (20.5%)	476 (63.9%)	153 (56.7%)	864 (84.5%)	**<0.001**
Bone mineral density					0.559
0	450 (68.5%)	507 (69.5%)	171 (63.8%)	691 (68.8%)	
1	199 (30.3%)	217 (29.7%)	92 (34.3%)	304 (30.3%)	
2	8 (1.2%)	6 (0.8%)	5 (1.9%)	9 (0.9%)	

Values are *n* (%), mean±SD, or median (lower quartile, upper quartile).

BMI, body mass index; BMR, basal metabolic rate; CAD, coronary artery disease; DBP, diastolic blood pressure; FF, fat fraction; FMI, fat mass index; FFMI, Fat-Free Mass Index; PR, pulse rate; SBP, systolic blood pressure; SMI, Skeletal Muscle Mass Index; VFA, visceral fat area.*P* are from two-sided tests and compared to a significance level of 5%.

According to BMI status, participants were categorized into normal weight (BMI between 18.5 and 24 kg/m^2^) and overweight/obese (BMI ≥ 24 kg/m^2^). Participants with two or more of the following four components were considered metabolically unhealthy (1): increased BP: systolic blood pressure (SBP) ≥ 130 mmHg or diastolic blood pressure (DBP) ≥ 85 mmHg or use of antihypertensive drugs; (2) Abnormal glucose metabolism: Fasting blood glucose (FBG) ≥ 5.6 mmol/L or glycosylated hemoglobin (HbA1c) ≥ 6.0% or use of hypoglycemic drugs; (3) elevated triglycerides (TG) level: TG ≥ 1.7 mmol/L or use of lipid-lowering drugs; (4) Low high-density lipoprotein cholesterol (HDL-C) (HDL-C < 1.03 mmol/L for men or < 1.29 mmol/L for women) or use of lipid-lowering medications.

Based on the combination of BMI and metabolic status, the subjects were divided into four metabolic phenotypes: (1) metabolically healthy normal weight (MHNW, BMI between 18.5 and 23.9 kg/m^2^ and zero or one metabolic abnormality); (2) metabolically healthy overweight or obesity (MHO, BMI ≥ 24 kg/m^2^ and zero or one metabolic abnormality); (3) metabolically unhealthy normal weight (MUNW, BMI between 18.5 and 23.9 kg/m^2^ and two or more metabolic abnormalities); and (4) metabolically unhealthy overweight or obesity (MUO, BMI ≥ 24 kg/m^2^ and two or more metabolic abnormalities).

### Statistical analysis

Statistical analysis was performed using R software (version 4.3.3). For continuous variables, normality tests were conducted, if the data follow a normal distribution, continuous variables are presented as mean ± SD and compared using Student’s t-test, while non-normally distributed variables are presented as median (inter-quartile range, IQR) and compared using the Mann–Whitney U test. Categorical variables are expressed as numbers and percentages, and the chi-squared (Χ^2^) test was used for comparison between groups. Spearman’s rank correlation method was used to analyze the relationship between indicators.

Cluster analysis was performed based on Age, Height, Weight, BMI, FF, VFA, BMR, SMI, FFMI, FMI, and InBody score in both male and female populations separately. Before clustering, the quantitative data are standardized according to the mean and standard deviation. In SPSS version 26.0, the TwoStep clustering method is used to perform cluster analysis on the standardized data with a mean of 0 and a standard deviation of 1. No dimensionality reduction was performed, and clustering was performed in the original space. The first step of the TwoStep clustering method is to estimate the optimal number of clusters based on the silhouette coefficient of 0.4, and the second step is hierarchical clustering. The analysis uses log-likelihood as the distance metric and uses the Schwarz-Bayesian criterion for clustering, with the number of clusters ranging from 2 to 15. And the best number of clusters was automatically decided by the algorithm.

A prediction model was developed based on the metabolic phenotype and clustering results in male and female subgroups, with the presence of underlying diseases as the outcome. The area under the curve was used to evaluate the model’s performance.

## Results

### Participants characteristics

A total of 3,589 participants were initially enrolled. However, only 2,716 patients with complete data were categorized into MHNW, MHO, MUNW, and MUO groups. After comparison, we found that all demographic characteristics and InBody examination indices were significantly different among the four groups (*p* < 0.001), and only 5 (ALB/GLB, LDL-C, TSH, FT4, and AFP) out of 30 hematological indicators were not significantly different among the four metabolic subgroups. As for medical history data, the prevalence of hypertension, diabetes, hyperuricemia, and fatty liver was lower in MHNW participants (all *p* < 0.05) (Table 1; Supplementary Table 1).

### Correlation analysis

The correlation analysis between weight, height, BMI, body indexes, and other patients’ characteristics is shown in Table 2; Supplementary Table 2. The results also revealed that only Cr (*r* = 0.054, *p* = 0.001), ALB/GLB (*r* = 0.126, *p* < 0.001), and HDL-C (*r* = 0.106, *p* < 0.001) showed a positive association with InBody score, which is the most commonly used InBody index.

**Table 2:** Correlations between InBody indexes and patient characteristics.

InBody indexes	Height	Weight	BMI	FF	VFA	BMR	SMI	FFMI	FMI	InBody score
Hypertension	0.045 (0.008)	0.189 (<0.001)	0.229 (<0.001)	0.075 (<0.001)	0.173 (<0.001)	0.132 (<0.001)	0.170 (<0.001)	0.182 (<0.001)	0.158 (<0.001)	−0.103 (<0.001)
Diabetes	0.055 (0.001)	0.065 (<0.001)	0.049 (0.003)	−0.032 (0.052)	0.028 (0.098)	0.066 (<0.001)	0.060 (<0.001)	0.071 (<0.001)	−0.002 (0.900)	−0.042 (0.011)
Hyperlipidemia	0.041 (0.014)	0.108 (<0.001)	0.114 (<0.001)	0.014 (0.414)	0.060 (<0.001)	0.092 (<0.001)	0.107 (<0.001)	0.117 (<0.001)	0.061 (<0.001)	−0.022 (0.186)
CAD	0.033 (0.049)	0.045 (0.007)	0.036 (0.029)	−0.009 (0.578)	0.021 (0.210)	0.048 (0.004)	0.048 (0.004)	0.049 (0.003)	0.010 (0.551)	−0.015 (0.363)
Coronary artery surgery	0.028 (0.096)	0.023 (0.165)	0.013 (0.429)	−0.006 (0.729)	0.011 (0.526)	0.025 (0.136)	0.021 (0.199)	0.016 (0.344)	0.004 (0.804)	−0.010 (0.552)
Hyperuricemia	0.076 (<0.001)	0.096 (<0.001)	0.073 (<0.001)	−0.003 (0.872)	0.048 (0.004)	0.095 (<0.001)	0.091 (<0.001)	0.091 (<0.001)	0.033 (0.050)	−0.034 (0.039)
Gout	0.044 (0.008)	0.045 (0.007)	0.027 (0.110)	−0.039 (0.019)	<0.001 (0.984)	0.059 (<0.001)	0.057 (0.001)	0.060 (<0.001)	−0.014 (0.389)	0.015 (0.382)
Urolithiasis	−0.005 (0.769)	−0.008 (0.646)	−0.004 (0.793)	−0.006 (0.730)	−0.008 (0.613)	−0.003 (0.874)	0.003 (0.874)	0.005 (0.757)	−0.001 (0.950)	−0.013 (0.434)
Nephritis or nephrotic syndrome	−0.004 (0.799)	−0.021 (0.201)	−0.022 (0.195)	−0.002 (0.927)	−0.008 (0.612)	−0.017 (0.295)	−0.019 (0.260)	−0.023 (0.176)	−0.008 (0.648)	0.001 (0.972)
Hypothyroidism	−0.019 (0.251)	−0.002 (0.911)	0.009 (0.597)	0.036 (0.030)	0.036 (0.033)	−0.020 (0.236)	−0.012 (0.461)	−0.017 (0.312)	0.030 (0.071)	−0.013 (0.447)
SBP	0.086 (<0.001)	0.279 (<0.001)	0.322 (<0.001)	0.130 (<0.001)	0.265 (<0.001)	0.198 (<0.001)	0.238 (<0.001)	0.251 (<0.001)	0.239 (<0.001)	−0.151 (<0.001)
DBP	0.139 (<0.001)	0.334 (<0.001)	0.349 (<0.001)	0.091 (<0.001)	0.242 (<0.001)	0.265 (<0.001)	0.306 (<0.001)	0.314 (<0.001)	0.221 (<0.001)	−0.139 (<0.001)
PR	−0.026 (0.128)	−0.027 (0.105)	−0.013 (0.424)	0.086 (<0.001)	0.049 (0.003)	−0.060 (<0.001)	−0.067 (<0.001)	−0.073 (<0.001)	0.056 (0.001)	−0.082 (<0.001)
Fatty liver	0.217 (<0.001)	0.520 (<0.001)	0.550 (<0.001)	0.210 (<0.001)	0.423 (<0.001)	0.383 (<0.001)	0.429 (<0.001)	0.437 (<0.001)	0.396 (<0.001)	−0.213 (<0.001)
Bone mineral density	0.026 (0.128)	0.005 (0.749)	−0.016 (0.352)	−0.023 (0.174)	−0.001 (0.963)	0.010 (0.578)	0.012 (0.477)	−0.001 (0.955)	−0.019 (0.264)	−0.072 (<0.001)

Values are *r* (*p* value).*P*-values are from two-sided tests and compared to a significance level of 5%.

### Clustering analysis

**Figure 1:**
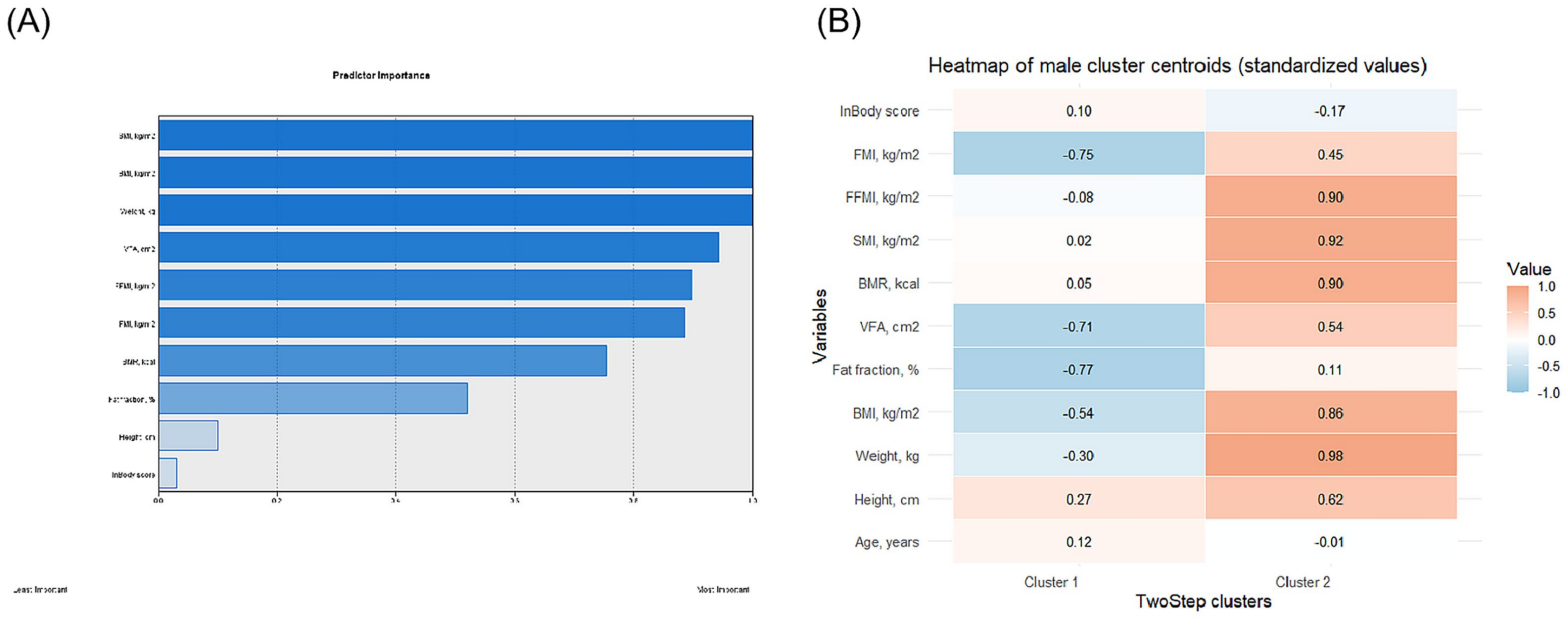
Visualization of feature importance **(A)** and distribution **(B)** in male participants.

For male participants, the whole cohort was split into two subgroups after clustering (Supplementary Table 3), and the feature importance and distribution of features are shown in Figure 1. The participants in cluster 2 were significantly higher, heavier, and showed higher BMI (all *p* < 0.001). The FF, VFA, BMR, SMI, FFMI, and FMI were also significantly higher (all *p* < 0.001) in cluster 2 participants, with the lower InBody score (68.0 [63.0; 73.0] vs. 69.0 [66.0; 73.0], *p* < 0.001), indicating the patients in cluster were under a worse metabolic status (Table 3).

**Table 3:** Clustering analysis in male participants.

Groups	Cluster 1 (*N* = 1,163)	Cluster 2 (*N* = 1,333)	*p*
Age, years	53.0 [42.0; 60.0]	51.0 [40.0; 58.0]	<0.001
Height, cm	170.0 [167.0; 174.5]	173.5 [170.0; 177.5]	<0.001
Weight, kg	68.1 [63.8; 71.7]	82.2 [78.0; 88.2]	<0.001
BMI, kg/m^2^	23.3 [21.9; 24.5]	27.4 [26.2; 29.0]	<0.001
FF, %	24.6 [21.2; 27.6]	29.8 [26.5; 33.1]	<0.001
VFA, cm^2^	72.5 [60.3; 84.1]	108.4 [93.9; 130.3]	<0.001
BMR, kcal	1477.0 [1407.0; 1535.5]	1634.0 [1550.0; 1727.0]	<0.001
SMI, kg/m^2^	7.5 [7.1; 7.7]	8.3 [8.0; 8.7]	<0.001
FFMI, kg/m^2^	17.5 [16.7; 18.2]	19.4 [18.6; 20.3]	<0.001
FMI, kg/m^2^	5.7 [4.7; 6.6]	8.2 [7.1; 9.4]	<0.001
InBody score	69.0 [66.0; 73.0]	68.0 [63.0; 73.0]	<0.001

Values are mean±SD, or median (lower quartile, upper quartile).*P* are from two-sided tests and compared to a significance level of 5%.

**Figure 2:**
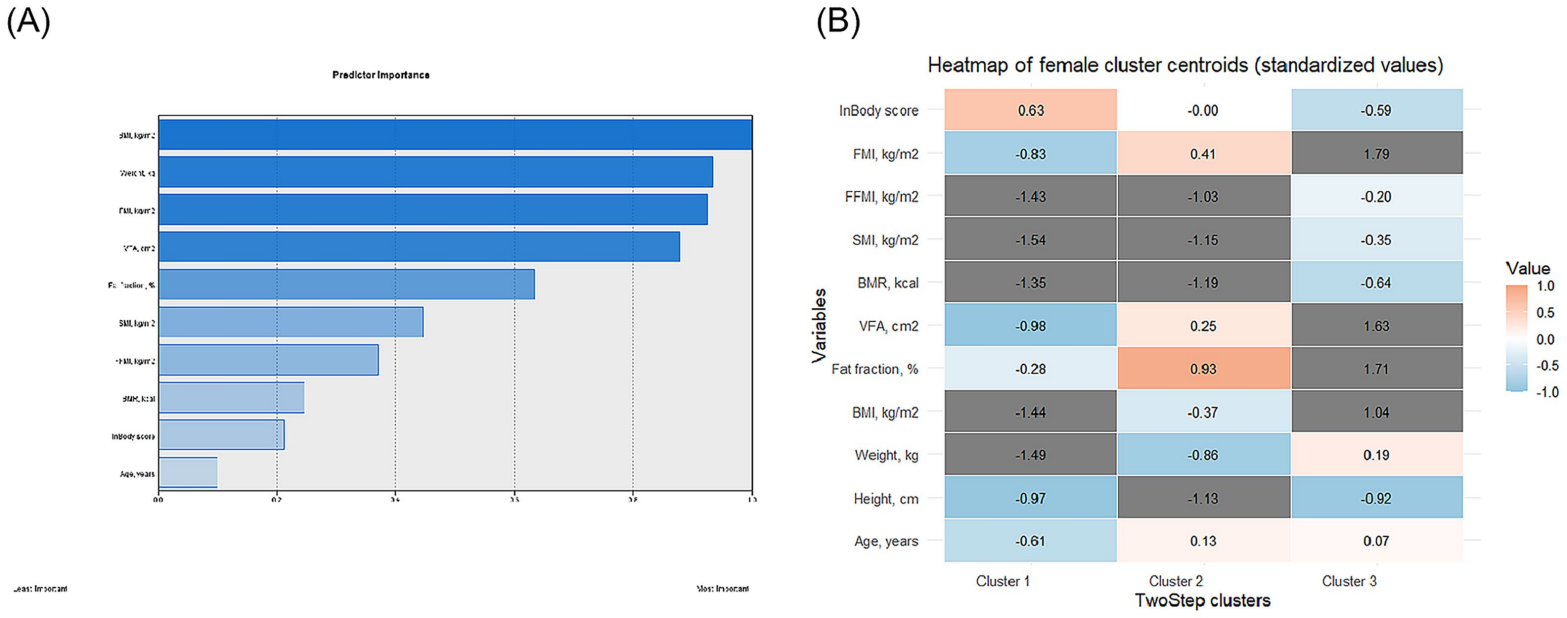
Visualization of feature importance **(A)** and distribution **(B)** in female participants.

The female participants were clustered into three subgroups (Supplementary Table 4), and the feature importance and distribution of features are shown in Figure 2. Similar to participants in male subgroups, there were significant differences in characteristics across participants in the three clusters regarding age, height, weight, and BMI (all *p* < 0.001). Moreover, the three female subgroups also showed a significant difference regarding the InBody score (cluster 1:72.0 [70.0; 75.0]; cluster 2: 69.0 [66.0; 71.0]; and cluster 3: 65.5 [62.0; 69.0], *p* < 0.001). Specifically, similar to male participants, across the three clusters, with the InBody score decreased, the FF, VFA, BMR, SMI, FFMI, and FMI increased accordingly (all *p* < 0.001) (Table 4).

**Table 4:** Clustering analysis in female participants.

Groups	Cluster 1 (*N* = 344)	Cluster 2 (*N* = 565)	Cluster 3 (*N* = 184)	*p*
Age, years	40.0 [32.0; 51.0]	53.0 [44.0; 59.0]	52.0 [43.0; 59.0]	<0.001
Height, cm	160.0 [157.0; 164.0]	159.0 [156.0; 162.5]	161.0 [157.0; 165.0]	<0.001
Weight, kg	52.0 [48.7; 54.8]	59.8 [57.1; 62.9]	72.6 [69.3; 76.7]	<0.001
BMI, kg/m^2^	20.1 [19.2; 21.0]	23.5 [22.6; 24.8]	28.2 [27.0; 29.7]	<0.001
Fat fraction, %	27.3 ± 4.3	34.9 ± 3.5	39.7 ± 4.3	<0.001
VFA, cm^2^	62.0 [53.5; 70.8]	103.1 [89.4; 118.2]	149.3 [132.6; 169.3]	<0.001
BMR, kcal	1180.0 [1124.5; 1238.0]	1211.0 [1164.0; 1262.0]	1329.0 [1263.0; 1384.5]	<0.001
SMI, kg/m^2^	5.8 ± 0.5	6.2 ± 0.4	7.0 ± 0.5	<0.001
FFMI, kg/m^2^	14.5 [13.7; 15.4]	15.4 [14.7; 16.0]	17.1 [16.4; 17.9]	<0.001
FMI, kg/m^2^	5.5 [4.8; 6.3]	8.2 [7.5; 8.9]	11.1 [10.1; 12.5]	<0.001
InBody score	72.0 [70.0; 75.0]	69.0 [66.0; 71.0]	65.5 [62.0; 69.0]	<0.001

Values are mean±SD, or median (lower quartile, upper quartile).*P* are from two-sided tests and compared to a significance level of 5%.

After taking clustering results and metabolic phenotypes into consideration simultaneously, we found that most male patients in cluster 2 were overweight or obese (99.6%), and the MUO took the largest proportion (61.2%), while among cluster 1 male participants, the distribution of patients with four metabolic phenotypes was relatively close. 39.1, 19.8, 21.0, and 20.2% for MHNW, MHO, MUNW, and MUO, respectively (Table 5).

**Table 5:** Metabolic phenotype in male participants at different clusters.

Groups	Cluster 1 (*N* = 916)	Cluster 2 (*N* = 1,138)	*p*
Metabolic phenotypes			<0.001
MHNW	358 (39.1%)	3 (0.3%)	
MHO	181 (19.8%)	438 (38.5%)	
MUNW	192 (21.0%)	1 (0.1%)	
MUO	185 (20.2%)	696 (61.2%)	

Values are *n* (%).*P* are from two-sided tests and compared to a significance level of 5%.

Table 6 shows the distribution of metabolic phenotypes and clustering results in the female population. Similar to male subgroups, cluster 3 was mainly composed of overweight or obese participants (98.4%), while Cluster 1 was composed only with normal weight participants (100.0%), and Cluster 2 participants exhibited a relatively stable composition of patients with different metabolic phenotypes, more specific, 45.4, 20.6, 15.0, and 18.9% for MHNW, MHO, MUNW, and MUO, respectively (Table 6).

**Table 6:** Metabolic phenotype in female participants at different clusters.

Groups	Cluster 1 (*N* = 175)	Cluster 2 (*N* = 359)	Cluster 3 (*N* = 128)	*p*
Metabolic phenotypes				<0.001
MHNW	152 (86.9%)	163 (45.4%)	2 (1.6%)	
MHO	0 (0.0%)	74 (20.6%)	53 (41.4%)	
MUNW	23 (13.1%)	54 (15.0%)	0 (0.0%)	
MUO	0 (0.0%)	68 (18.9%)	73 (57.0%)	

Values are *n* (%).*P* are from two-sided tests and compared to a significance level of 5%.

### Prevalence of metabolic diseases in sex-dimorphic clustering subgroups

For male participants, the prevalence of hypertension (29.4% vs. 21.2%, *p* < 0.001) and hyperlipidemia (8.4% vs. 4.9%, *p* = 0.001) was significantly higher in the cluster 2 subgroup (Table 7). While in female participants, a significantly higher prevalence of hypertension (22.8%) and diabetes (4.9%) was found in cluster 3, and the participants in cluster 1 had the lowest prevalence of hypertension (3.2%) and diabetes (1.2%) (*p* values were < 0.001 and 0.021, respectively) (Table 8).

**Table 7:** Prevalence of metabolic diseases in male participants at different clusters.

Groups	Cluster 1 (*N* = 1,163)	Cluster 2 (*N* = 1,333)	*p*
Hypertension	247 (21.2%)	392 (29.4%)	**<0.001**
Diabetes	115 (9.9%)	106 (8.0%)	0.104
Hyperlipidemia	57 (4.9%)	112 (8.4%)	**0.001**
CAD	17 (1.5%)	21 (1.6%)	0.946
Hyperuricemia	32 (2.8%)	50 (3.8%)	0.199
Gout	16 (1.4%)	18 (1.4%)	1.000
Urolithiasis	10 (0.9%)	7 (0.5%)	0.441
Nephritis or nephrotic syndrome	1 (0.1%)	0 (0.0%)	0.466
Hypothyroidism	1 (0.1%)	5 (0.4%)	0.225

Values are *n* (%).*P* are from two-sided tests and compared to a significance level of 5%.

**Table 8:** Prevalence of metabolic diseases in female participants at different clusters.

Groups	Cluster 1 (*N* = 344)	Cluster 2 (*N* = 565)	Cluster 3 (*N* = 184)	*p*
Hypertension	11 (3.2%)	62 (11.0%)	42 (22.8%)	**<0.001**
Diabetes	4 (1.2%)	10 (1.8%)	9 (4.9%)	**0.021**
Hyperlipidemia	6 (1.7%)	10 (1.8%)	8 (4.3%)	0.114
CAD	1 (0.3%)	0 (0.0%)	0 (0.0%)	0.483
Hyperuricemia	0 (0.0%)	1 (0.2%)	0 (0.0%)	1
Gout	0 (0.0%)	0 (0.0%)	0 (0.0%)	-
Urolithiasis	1 (0.3%)	2 (0.4%)	0 (0.0%)	1
Nephritis or nephrotic syndrome	0 (0.0%)	1 (0.2%)	0 (0.0%)	1
Hypothyroidism	1 (0.3%)	6 (1.1%)	2 (1.1%)	0.444

Values are *n* (%).*P* are from two-sided tests and compared to a significance level of 5%.

### Prediction of underlying diseases using metabolic phenotype and clustering results

**Figure 3:**
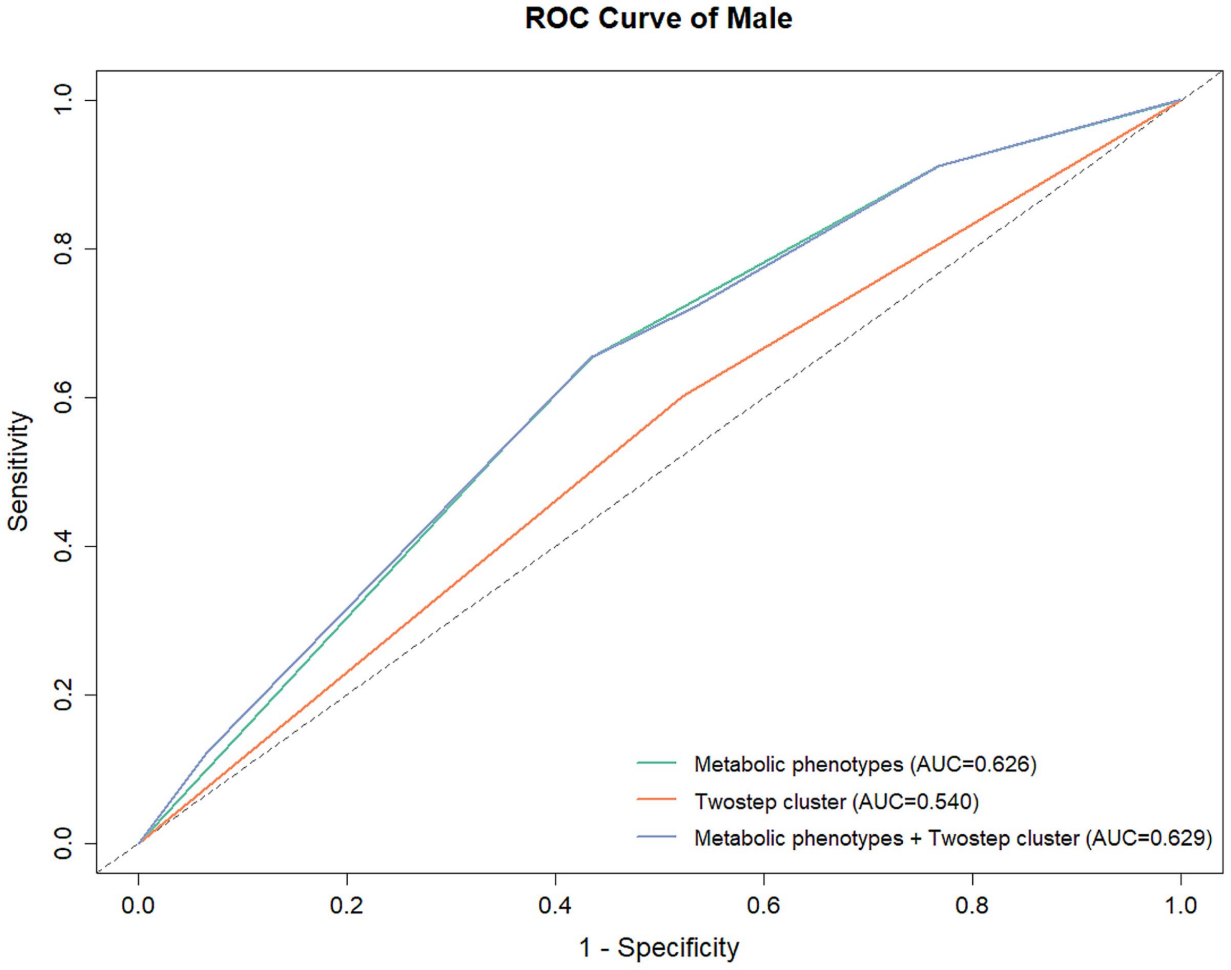
ROC for the prediction of underlying diseases in male participants.

**Figure 4:**
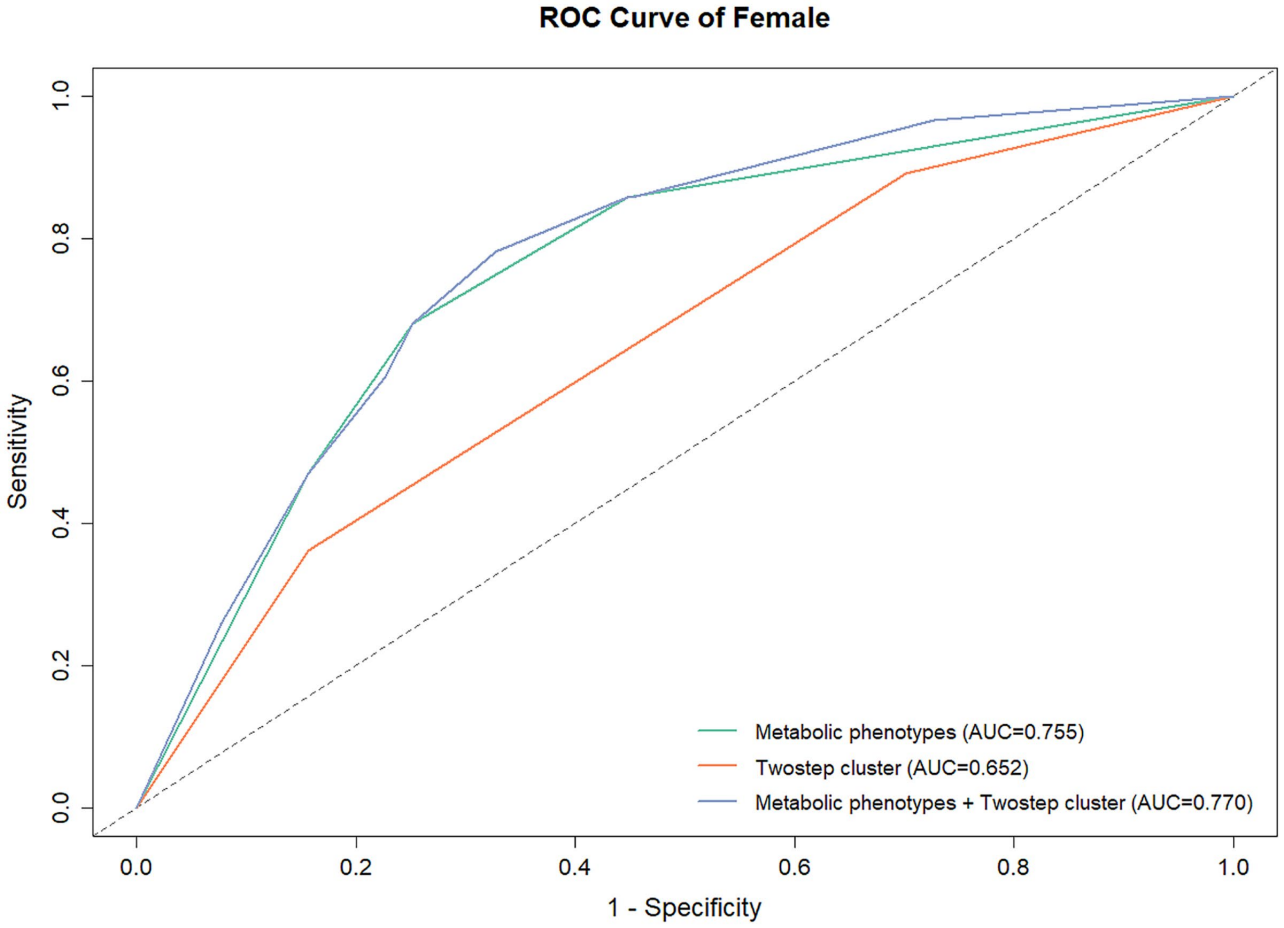
ROC for the prediction of underlying diseases in female participants.

In male participants, the AUCs were 0.629 (95% CI: 0.605–0.652), 0.626 (95% CI: 0.603–0.648), and 0.547 (95% CI: 0.527–0.567) for metabolic phenotypes + clustering results, metabolic phenotypes, and clustering results, respectively (Table 9; Figure 3). While in female participants, the AUCs were 0.770 (95% CI: 0.726–0.813), 0.755 (95% CI: 0.710–0.799), and 0.651 (95% CI: 0.610–0.692) for metabolic phenotypes + clustering results, metabolic phenotypes, and clustering results, respectively (Table 9; Figure 4). Indicating that the prediction model performance was better in the female group.

**Table 9:** Prediction model performance.

Characteristic		AUC	95% CI	*p*	Sensitivity	Specificity
Male	Metabolic phenotypes	0.626	0.603–0.648	<0.001	0.654	0.564
	TwoStep cluster	0.547	0.527–0.567	<0.001	0.594	0.499
	Metabolic phenotypes+ TwoStep cluster	0.629	0.605–0.652	<0.001	0.654	0.565
Female	Metabolic phenotypes	0.755	0.710–0.799	<0.001	0.681	0.748
	TwoStep cluster	0.651	0.610–0.692	<0.001	0.872	0.344
	Metabolic phenotypes+ TwoStep cluster	0.770	0.726–0.813	<0.001	0.782	0.672

AUC, area under the curve.

## Discussion

In this population-based study, we found that the InBody-based clustering analysis could classify people into different subgroups, which might indicate specific obese people with a higher risk of metabolic diseases. The findings of this study provide new ideas for managing metabolic diseases at a population level.

Body composition estimation is an important component in various professional and medical settings, and could be used to evaluate obesity, sarcopenia, and osteoporosis (19–21). Katahira et al. showed that estimated VFA is a better index for the evaluation of waist circumference for the diagnosis of MetS in central obesity patients (22), while Sarahi et al. developed a metabolic age prediction algorithm based on BMR and showed that the proposed method could facilitate the prediction of metabolic syndrome (23). however in the previous mentioned studies, BMR or VFA was evaluated using conventional techniques, while in some recent studies, the InBody was adopted and used in the evaluation of metabolic syndrome, Mohammad et al. revealed that decreased BMR, FFM, and total body water were associated with a high-antioxidant diet in normal-weight metabolic syndrome patients (24); Habib et al. collected InBody examination based body composition information in 360 obesity and overweight women, and they found that FFMI were different in participants with different nutrient patterns (25). However, to our knowledge, no study used the InBody indexes for the machine learning-based clustering of participants and explored the clustering results with the metabolic phenotypes. In the present study, we did the clustering analysis in male and female separately for the differences of body composition between different sexes, the body composition could be affected by hormone levels (26), eating habit (27), and developmental differences (28), the results also confirmed this hypothesis, we found clustering algorithm categorized male and female participants into two and three subgroups, and the obesity/overweight rate in male participants of cluster 2 reached 99.6%, while the obesity/overweight rate in female participants of cluster 3 reached 98.4%, indicating that BMI > 24 is a significant characteristic for the automatic clustering algorithm, the feature importance also revealed that BMI was the most importance feature in both male and female subgroups. We also noticed that female cluster 2 and male cluster 1, female cluster 3 and male cluster 2 showed similar metabolic phenotype composition, while female participants have an additional cluster 1 with only normal-weight participants included (86.9% MHNW and 13.1% MUNW), indicating that normal-weight female patients may represent a group of participants in the Chinese population, which is also in alignment with the results of a large-scale Chinese cohort study (29), we also noticed that the prevalence of hypertension (3.2% vs. 11.0 and 22.8%, *p* < 0.001) and diabetes (1.2% vs. 1.8 and 4.9%, *p* = 0.021) were also lower in cluster 1 female, which was also in agreement with several previous studies, Xu reported that overweight obese Chinese participants had a higher risk of developing diabetes compared to normal weight non obese participants (30), while Ren et al. reported that overweight-obese subjects with central obesity demonstrated the highest risk of hypertension compared to normal weight but no central obesity participants (31). The findings of the present study directly revealed that InBody examination results are different in both sexes, and the differences could further help differentiate participants into 2 or more subgroups, which is of great importance in a clinical scenario, Trempe et al. found that women presented a higher prevalence of abdominal obesity and elevated visceral fat level (32), however, when considering subgroups, the results could be different, and the subgroup classification provided a more delicate risk stratification and health status evaluation tool.

The association between hematological indicators and metabolic phenotypes is another important research topic for a better understanding of individualized health status. Several previous studies have revealed the specific associations between hematological indicators and metabolic phenotypes, both Feng and Zhao focused on serum uric acid and overweight/obese metabolic phenotypes, their results showed that hyperuricemia was positively associated with MHO and MU subjects (33, 34); while Ferreira et al. explored the relation between cytokine levels and different metabolic phenotypes and showed MUO subjects had a higher risk for increased cytokine levels (35). However, the previously mentioned studies only concentrated on limited hematological features, while in the present study, we analyzed the association between body index and about 50 characteristics, including both medical history and hematological indicators. Our results also reflect that a higher InBody score correlates with a better hematological status. Except for the hematological indicators, age, BMI and other basic characteristics were also important for the metabolic phenotypes and InBody clustering results, Murthy revealed that metabolic phenotype could refines cardiovascular risk evaluation in young adults (36), while Cho et al. showed an age-related metabolic derangement through CT body composition method (37), while in the present study, we included age in the clustering analysis, which could provide a more comprehensive spectrum of InBody-based cluster results.

Apart from hematological features and InBody indexes, this study also evaluated the associations between metabolic disease prevalence and clustering results. Although many studies have assessed the underlying relation between metabolic diseases and metabolic phenotypes, Zhao revealed that obesity was significantly associated with renal disease, while adding metabolic unhealthy status further increased the risk (38); Kim et al. stressed that the metabolic milieu beyond obesity could play a significant role in patients with hepatic fibrosis (39). The present study also showed that the prevalence of hypertension, diabetes, hyperlipidemia, and hyperuricemia was significantly different between patients of the four metabolic phenotypes. However, after a sex-dimorphic cluster analysis, only hypertension and hyperlipidemia remained significant in the male population, for females, the difference in prevalence was only observed in hypertension and diabetes. These results indicated that different disease prevalence may have a sex-specific and metabolism-dependent pattern. However, the robustness of this study was limited by the low prevalence of these diseases, and large-scale comorbidities data should be used to further validate our results. Still, these findings could provide more details for the understanding of metabolic diseases.

However, this study still had several limitations. Firstly, although more than three thousand participants were initially enrolled, this study was still a retrospective single-center study which unavoidably affected by the limitation of the study population, further external validation is required before the application of the study results. Secondly, the study only analyzed participants with normal weight and obesity/overweight, participants with reduced weight were excluded from the present study to avoid confounding, since low body weight often reflects distinct underlying illnesses or etiologies. Nevertheless, future studies should include participants with reduced weight for better generalization ability. Thirdly, the machine learning based clustering method could be difficult to understand for some clinicians, a more acceptable interpretation of the clustering results was in urgent need. Furthermore, although the clustering method did not require factors without multicollinearity, the highly correlated variables may amplify the weight of characteristics, which could be one of the strategies for improving the algorithm in the future. Finally, the present study focused on the InBody test results, due to the retrospective nature of this study, some basic information including smoking status, activity levels were not documented for all participants, moreover, the follow-up data was not obtained, inclusion of follow-up data and complete basic characteristics may facilitate better understanding of the cluster results in future studies.

## Conclusion

This study showed that the InBody-based clustering analysis could classify the populations into different subgroups, indicating that InBody examination should be performed in a clinical scenario, and specific obese populations with a higher risk of metabolic diseases should receive more frequent health check-ups.

## Data Availability

The original contributions presented in the study are included in the article/Supplementary material, further inquiries can be directed to the corresponding authors.
