# Supplementary material for: A body composition-based clustering study and its association with metabolic phenotypes among the general population in China
Source: Front Nutr. 2025 Oct 31;12:1636849. doi: 10.3389/fnut.2025.1636849 (PMC12616634; doi:10.3389/fnut.2025.1636849)
Supplement: Supplementary file 1 [file Table_1.DOCX]

Supplementary Table 1. Baseline laboratory examination results of participants.

| Metabolic phenotypes | MHNW (N=678) | MHO (N=746) | MUNW (N=270) | MUO (N=1022) | P |
| --- | --- | --- | --- | --- | --- |
| BUN | 5.1 [ 4.3; 6.0] | 5.5 [ 4.7; 6.4] | 5.5 [ 4.7; 6.3] | 5.5 [ 4.7; 6.4] | **<0.001** |
| Cr | 63.5 [53.0;75.0] | 71.0 [62.0;79.0] | 67.0 [56.0;78.0] | 70.0 [60.0;78.0] | **<0.001** |
| UA | 313.6 [261.5;363.0] | 367.8 [315.1;422.5] | 353.2 [300.2;408.9] | 390.2 [335.7;443.7] | **<0.001** |
| GFR | 110.2 [97.9;124.8] | 105.7 [94.6;119.8] | 107.0 [92.3;123.1] | 107.4 [94.1;124.7] | **0.002** |
| ALT | 17.0 [13.0;24.0] | 23.0 [17.0;32.0] | 22.0 [16.0;29.0] | 27.0 [20.0;41.0] | **<0.001** |
| AST | 20.0 [17.0;23.0] | 21.0 [18.0;25.0] | 21.0 [19.0;25.0] | 23.0 [18.0;28.0] | **<0.001** |
| GGT | 19.0 [14.0;28.0] | 26.0 [18.0;41.0] | 29.0 [19.0;46.0] | 37.0 [25.0;59.0] | **<0.001** |
| ALP | 68.0 [57.0;82.0] | 70.0 [59.0;84.0] | 74.0 [63.0;87.0] | 73.0 [62.0;88.0] | **<0.001** |
| LDH | 170.0 [152.0;187.0] | 178.0 [161.0;197.0] | 176.0 [160.0;194.0] | 178.0 [160.0;199.0] | **<0.001** |
| CHE | 347.0 [304.0;393.0] | 389.0 [342.0;437.0] | 394.0 [342.0;447.0] | 421.0 [371.0;471.0] | **<0.001** |
| LAP | 48.0 [44.0;54.0] | 51.0 [46.0;56.0] | 51.0 [46.0;57.0] | 53.0 [47.0;59.0] | **<0.001** |
| TP | 72.7 [70.4;75.2] | 73.2 [70.8;75.7] | 73.9 [71.3;76.7] | 73.7 [71.1;76.1] | **<0.001** |
| ALB | 46.0 ± 2.5 | 46.2 ± 2.4 | 46.5 ± 2.7 | 46.4 ± 2.3 | **<0.001** |
| GLB | 26.8 [24.8;28.6] | 26.9 [24.7;28.9] | 27.2 [25.4;29.1] | 27.2 [25.3;29.2] | **0.001** |
| ALB/GLB | 1.7 [ 1.6; 1.8] | 1.7 [ 1.6; 1.9] | 1.7 [ 1.6; 1.8] | 1.7 [ 1.6; 1.8] | 0.293 |
| TB | 12.0 [ 9.6;15.2] | 12.8 [10.1;16.4] | 12.4 [ 9.5;15.8] | 12.0 [ 9.6;15.3] | **0.001** |
| IBIL | 8.9 [ 7.0;11.2] | 9.2 [ 7.3;12.1] | 9.1 [ 6.9;11.6] | 8.8 [ 7.0;11.3] | **0.023** |
| DBIL | 3.1 [ 2.4; 4.1] | 3.6 [ 2.7; 4.8] | 3.2 [ 2.3; 4.1] | 3.2 [ 2.5; 4.1] | **<0.001** |
| TBA | 3.0 [ 2.0; 4.5] | 3.2 [ 2.2; 5.1] | 3.6 [ 2.6; 5.7] | 3.8 [ 2.6; 5.8] | **<0.001** |
| TC | 5.0 [ 4.4; 5.6] | 4.9 [ 4.3; 5.5] | 5.1 [ 4.4; 5.9] | 5.0 [ 4.3; 5.6] | **0.025** |
| TG | 1.0 [ 0.8; 1.3] | 1.2 [ 0.9; 1.6] | 1.9 [ 1.3; 2.5] | 2.2 [ 1.7; 3.1] | **<0.001** |
| HDL-C | 1.5 [ 1.3; 1.7] | 1.3 [ 1.1; 1.5] | 1.2 [ 1.0; 1.5] | 1.1 [ 0.9; 1.3] | **<0.001** |
| LDL-C | 3.1 [ 2.5; 3.7] | 3.2 [ 2.6; 3.7] | 3.2 [ 2.6; 3.9] | 3.1 [ 2.6; 3.7] | 0.165 |
| HbA1c | 5.6 [ 5.4; 5.8] | 5.6 [ 5.4; 5.8] | 6.0 [ 5.6; 6.5] | 6.0 [ 5.7; 6.4] | **<0.001** |
| Glu | 5.0 [ 4.6; 5.3] | 5.1 [ 4.8; 5.4] | 5.7 [ 5.1; 6.5] | 5.7 [ 5.2; 6.6] | **<0.001** |
| TSH | 1.9 [ 1.3; 2.7] | 1.9 [ 1.3; 2.7] | 2.0 [ 1.4; 3.0] | 2.0 [ 1.4; 2.8] | 0.470 |
| FT4 | 11.4 [10.3;12.6] | 11.2 [10.2;12.5] | 11.2 [10.2;12.5] | 11.3 [10.2;12.4] | 0.432 |
| FT3 | 5.2 [ 4.8; 5.7] | 5.4 [ 5.0; 5.9] | 5.4 [ 4.9; 5.8] | 5.5 [ 5.1; 5.9] | **<0.001** |
| AFP | 3.2 [ 2.3; 4.4] | 3.2 [ 2.3; 4.4] | 3.1 [ 2.4; 4.4] | 3.2 [ 2.5; 4.5] | 0.680 |
| CEA | 1.4 [ 0.8; 2.1] | 1.4 [ 0.9; 2.2] | 1.6 [ 1.0; 2.5] | 1.6 [ 1.0; 2.4] | **<0.001** |

AFP, Alpha-fetoprotein; ALB, albumin; ALP, alkaline phosphatase; ALT, Alanine aminotransferase; AST, Aspartate aminotransferase; BUN, Blood Urea Nitrogen; CEA, carcinoembryonic antigen; CHE, cholinesterase; Cr, creatinine; DBIL, direct bilirubin; FT4, Free thyroxine; FT3, free triiodothyronine; GFR, glomerular filtration rate; GGT, gamma-glutamyltransferase; GLB, globulin; Glu, glucose; HbA1c, Glycated haemoglobin; HDL-C, High density lipoprotein cholesterol; LAP, leucine aminopeptidase; IBIL, Indirect bilirubin; LDH, lactate dehydrogenase; LDL-C, Low density lipoprotein cholesterol; TB, total bilirubin; TP, total protein; TBA, total bile acid; TC, total cholesterol; TG, triglyceride; TSH, thyroidstimulatinghormone; UA, uric acid.

Supplementary Table 2. Correlations between InBody indexes and patient laboratory examination results.

|  | Height | Weight | BMI | FF | VFA | BMR | SMI | FFMI | FMI | InBody score |
| --- | --- | --- | --- | --- | --- | --- | --- | --- | --- | --- |
| Hypertension | 0.045 (0.008) | 0.189 (<0.001) | 0.229 (<0.001) | 0.075 (<0.001) | 0.173 (<0.001) | 0.132 (<0.001) | 0.170 (<0.001) | 0.182 (<0.001) | 0.158 (<0.001) | -0.103 (<0.001) |
| Diabetes | 0.055 (0.001) | 0.065 (<0.001) | 0.049 (0.003) | -0.032 (0.052) | 0.028 (0.098) | 0.066 (<0.001) | 0.060 (<0.001) | 0.071 (<0.001) | -0.002 (0.900) | -0.042 (0.011) |
| Hyperlipidemia | 0.041 (0.014) | 0.108 (<0.001) | 0.114 (<0.001) | 0.014 (0.414) | 0.060 (<0.001) | 0.092 (<0.001) | 0.107 (<0.001) | 0.117 (<0.001) | 0.061 (<0.001) | -0.022 (0.186) |
| CAD | 0.033 (0.049) | 0.045 (0.007) | 0.036 (0.029) | -0.009 (0.578) | 0.021 (0.210) | 0.048 (0.004) | 0.048 (0.004) | 0.049 (0.003) | 0.010 (0.551) | -0.015 (0.363) |
| Coronary artery surgery | 0.028 (0.096) | 0.023 (0.165) | 0.013 (0.429) | -0.006 (0.729) | 0.011 (0.526) | 0.025 (0.136) | 0.021 (0.199) | 0.016 (0.344) | 0.004 (0.804) | -0.010 (0.552) |
| Hyperuricemia | 0.076 (<0.001) | 0.096 (<0.001) | 0.073 (<0.001) | -0.003 (0.872) | 0.048 (0.004) | 0.095 (<0.001) | 0.091 (<0.001) | 0.091 (<0.001) | 0.033 (0.050) | -0.034 (0.039) |
| Gout | 0.044 (0.008) | 0.045 (0.007) | 0.027 (0.110) | -0.039 (0.019) | <0.001 (0.984) | 0.059 (<0.001) | 0.057 (0.001) | 0.060 (<0.001) | -0.014 (0.389) | 0.015 (0.382) |
| Urolithiasis | -0.005 (0.769) | -0.008 (0.646) | -0.004 (0.793) | -0.006 (0.730) | -0.008 (0.613) | -0.003 (0.874) | 0.003 (0.874) | 0.005 (0.757) | -0.001 (0.950) | -0.013 (0.434) |
| Nephritis or nephrotic syndrome | -0.004 (0.799) | -0.021 (0.201) | -0.022 (0.195) | -0.002 (0.927) | -0.008 (0.612) | -0.017 (0.295) | -0.019 (0.260) | -0.023 (0.176) | -0.008 (0.648) | 0.001 (0.972) |
| Hypothyroidism | -0.019 (0.251) | -0.002 (0.911) | 0.009 (0.597) | 0.036 (0.030) | 0.036 (0.033) | -0.020 (0.236) | -0.012 (0.461) | -0.017 (0.312) | 0.030 (0.071) | -0.013 (0.447) |
| SBP | 0.086 (<0.001) | 0.279 (<0.001) | 0.322 (<0.001) | 0.130 (<0.001) | 0.265 (<0.001) | 0.198 (<0.001) | 0.238 (<0.001) | 0.251 (<0.001) | 0.239 (<0.001) | -0.151 (<0.001) |
| DBP | 0.139 (<0.001) | 0.334 (<0.001) | 0.349 (<0.001) | 0.091 (<0.001) | 0.242 (<0.001) | 0.265 (<0.001) | 0.306 (<0.001) | 0.314 (<0.001) | 0.221 (<0.001) | -0.139 (<0.001) |
| PR | -0.026 (0.128) | -0.027 (0.105) | -0.013 (0.424) | 0.086 (<0.001) | 0.049 (0.003) | -0.060 (<0.001) | -0.067 (<0.001) | -0.073 (<0.001) | 0.056 (0.001) | -0.082 (<0.001) |
| Fatty liver | 0.217 (<0.001) | 0.520 (<0.001) | 0.550 (<0.001) | 0.210 (<0.001) | 0.423 (<0.001) | 0.383 (<0.001) | 0.429 (<0.001) | 0.437 (<0.001) | 0.396 (<0.001) | -0.213 (<0.001) |
| Bone mineral density | 0.026 (0.128) | 0.005 (0.749) | -0.016 (0.352) | -0.023 (0.174) | -0.001 (0.963) | 0.010 (0.578) | 0.012 (0.477) | -0.001 (0.955) | -0.019 (0.264) | -0.072 (<0.001) |

Values are r (p value).

Supplementary Table 3. TwoStep cluster for male participants.

|  | **Cluster** | **Schwarz's Bayesian Criterion (BIC)** | **BIC Change** | **Ratio of BIC Changes** | **Ratio of Distance Measures** |
| --- | --- | --- | --- | --- | --- |
| **Male,** **Silhouette = 0.4** | 1 | 19197.642 |  |  |  |
|  | **2** | **14896.616** | **-4301.026** | **1.000** | **2.507** |
|  | 3 | 13284.273 | -1612.343 | 0.375 | 1.435 |
|  | 4 | 12212.756 | -1071.517 | 0.249 | 1.401 |
|  | 5 | 11497.045 | -715.712 | 0.166 | 1.018 |
|  | 6 | 10796.610 | -700.435 | 0.163 | 1.428 |
|  | 7 | 10357.869 | -438.741 | 0.102 | 1.245 |
|  | 8 | 10039.263 | -318.606 | 0.074 | 1.058 |
|  | 9 | 9747.525 | -291.738 | 0.068 | 1.168 |
|  | 10 | 9522.347 | -225.178 | 0.052 | 1.441 |
|  | 11 | 9418.725 | -103.622 | 0.024 | 1.306 |
|  | 12 | 9379.712 | -39.013 | 0.009 | 1.072 |
|  | 13 | 9354.912 | -24.800 | 0.006 | 1.032 |
|  | 14 | 9336.256 | -18.656 | 0.004 | 1.033 |
|  | 15 | 9323.638 | -12.619 | 0.003 | 1.119 |

Supplementary Table 4. TwoStep cluster for female participants.

|  | **Cluster** | **Schwarz's Bayesian Criterion (BIC)** | **BIC Change** | **Ratio of BIC Changes** | **Ratio of Distance Measures** |
| --- | --- | --- | --- | --- | --- |
| **Female,** **Silhouette = 0.4** | 1 | 2645.489 |  |  |  |
|  | 2 | 1143.264 | -1502.225 | 1.000 | 1.417 |
|  | **3** | **128.052** | **-1015.212** | **0.676** | **2.078** |
|  | 4 | -280.590 | -408.642 | 0.272 | 1.450 |
|  | 5 | -514.715 | -234.125 | 0.156 | 1.090 |
|  | 6 | -716.879 | -202.164 | 0.135 | 1.263 |
|  | 7 | -844.888 | -128.009 | 0.085 | 1.222 |
|  | 8 | -921.754 | -76.866 | 0.051 | 1.313 |
|  | 9 | -943.655 | -21.901 | 0.015 | 1.189 |
|  | 10 | -937.657 | 5.998 | -0.004 | 1.371 |
|  | 11 | -891.643 | 46.013 | -0.031 | 1.131 |
|  | 12 | -833.094 | 58.550 | -0.039 | 1.002 |
|  | 13 | -774.377 | 58.716 | -0.039 | 1.031 |
|  | 14 | -712.792 | 61.585 | -0.041 | 1.125 |
|  | 15 | -640.939 | 71.854 | -0.048 | 1.025 |
